# 2-Phen­oxy­ethyl benzoate

**DOI:** 10.1107/S1600536813010878

**Published:** 2013-04-27

**Authors:** Mousa Al-Noaimi, Ismail Warad, Salim F. Haddad, Ahmad Husein, Rami Shareiah

**Affiliations:** a Department of Chemistry, Hashemite University, Zarqa 13115, Jordan; b Department of Chemistry, AN-Najah National University, Nablus, Palestinian Territories; c Department of Chemistry, The University of Jordan, Amman 11942, Jordan; d Department of Basic Science, Allied Medical Science College, Applied Science Private University, PO Box 166, Amman 11931, Jordan

## Abstract

In the title compound, C_15_H_14_O_3_, the dihedral angle between the benzene rings is 75.85 (7)°. In the crystal, centrosymmetrically related mol­ecules are weakly associated through pairs of inter­actions between a benzene ring and an O atom of the ester group [ring centroid⋯O = 3.952 (7) Å], and through pairs of inter­actions between the other benzene ring and an O atom of the phen­oxy group [ring centroid⋯O = 3.912 (7) Å], giving chains extending along [110].

## Related literature

For background information and related structures, see: Gandhi *et al.* (1995 ▶); Huang *et al.* (1996 ▶); Litera *et al.* (2006 ▶); Ruzicka *et al.* (2002 ▶); Sheehan & Umezaw (1973 ▶).
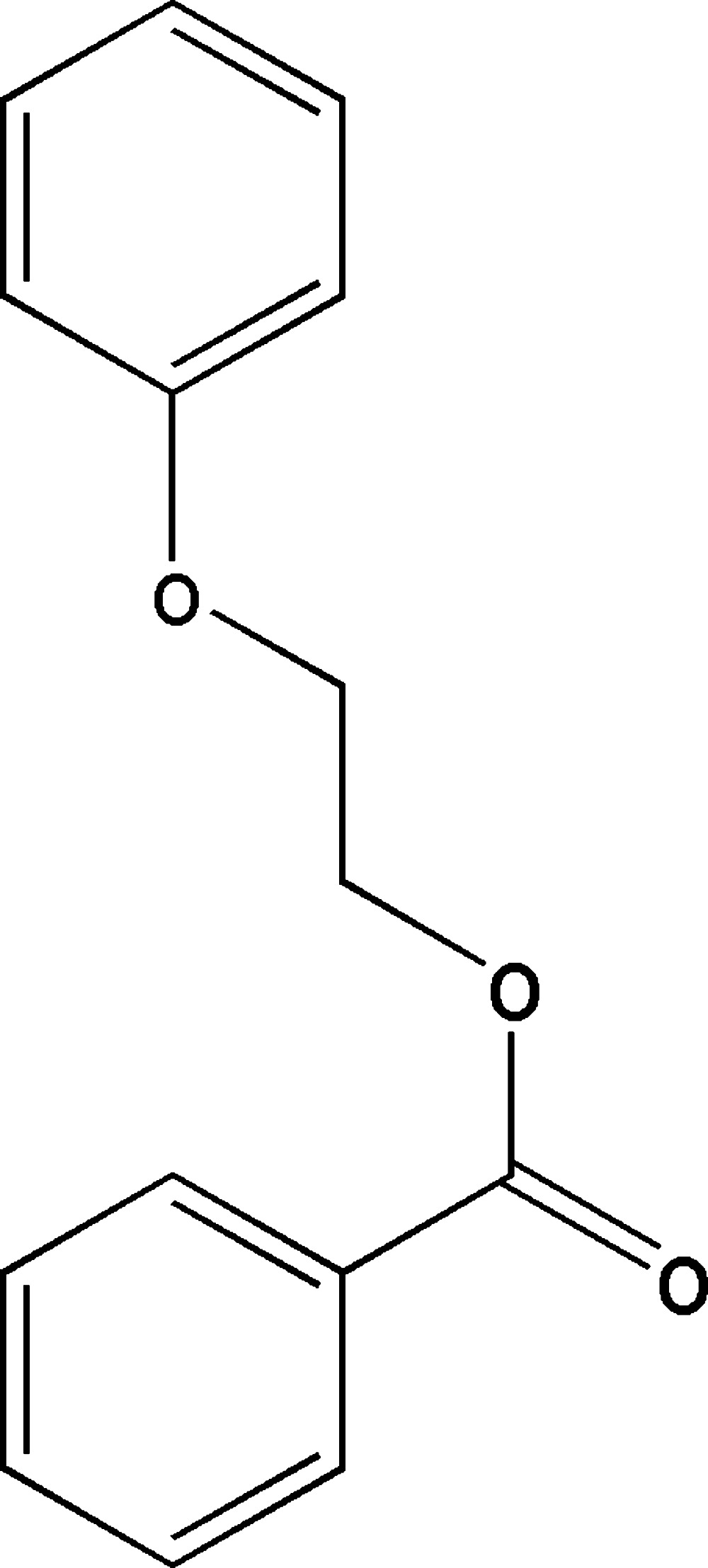


## Experimental

### 

#### Crystal data

C_15_H_14_O_3_
*M* *_r_* = 242.26
Monoclinic, 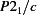

*a* = 9.4675 (10) Å
*b* = 10.1411 (10) Å
*c* = 13.7792 (12) Å
β = 103.895 (10)°
*V* = 1284.2 (2) Å^3^
*Z* = 4
Mo *K*α radiation
μ = 0.09 mm^−1^
*T* = 293 K
0.32 × 0.26 × 0.18 mm

#### Data collection

Oxford Diffraction Xcalibur Eos CCD-detector diffractometer
Absorption correction: analytical (*CrysAlis PRO*; Agilent, 2011 ▶) *T*_min_ = 0.995, *T*_max_ = 0.997
5089 measured reflections
2269 independent reflections
1479 reflections with *I* > 2σ(*I*)
*R*_int_ = 0.029

#### Refinement

*R*[*F* ^2^ > 2σ(*F* ^2^)] = 0.044
*wR*(*F* ^2^) = 0.130
*S* = 1.02
2269 reflections
164 parameters
H-atom parameters constrained
Δρ_max_ = 0.11 e Å^−3^
Δρ_min_ = −0.11 e Å^−3^

### 

Data collection: *CrysAlis PRO* (Agilent, 2011 ▶); cell refinement: *CrysAlis PRO*; data reduction: *CrysAlis PRO*; program(s) used to solve structure: *SHELXS97* (Sheldrick, 2008 ▶); program(s) used to refine structure: *SHELXL97* (Sheldrick, 2008 ▶); molecular graphics: *ORTEPIII* (Burnett & Johnson, 1996 ▶); software used to prepare material for publication: *SHELXL97*.

## Supplementary Material

Crystal structure: contains datablock(s) I, global. DOI: 10.1107/S1600536813010878/zs2256sup1.cif

Structure factors: contains datablock(s) I. DOI: 10.1107/S1600536813010878/zs2256Isup2.hkl

Supplementary material file. DOI: 10.1107/S1600536813010878/zs2256Isup3.cml

Additional supplementary materials:  crystallographic information; 3D view; checkCIF report

## Figures and Tables

**Fig. 1.**
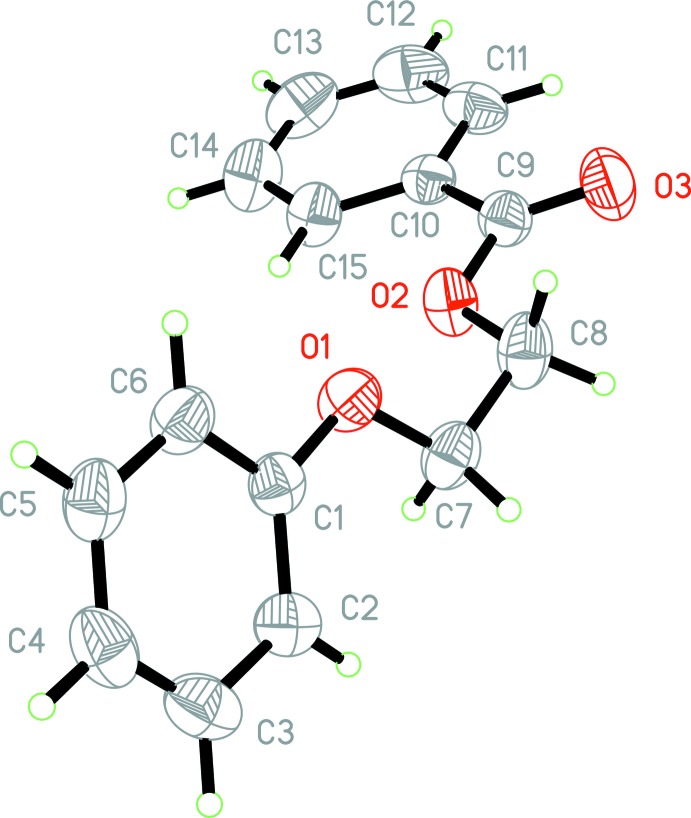
Molecular conformation and atom numbering scheme for the title compound. Displacement ellipsoids are drawn at the 30% probability level. Hydrogen atoms are represented as small spheres of arbitrary radii.

**Fig. 2.**
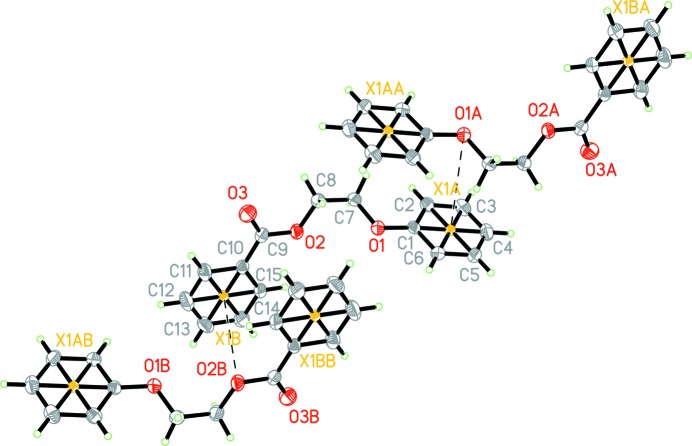
The chain structure showing the ring centroid···O associations [for symmetry code (A): -x, -y + 1, -z]; (B): -x + 1, -y, -z].

## supplementary crystallographic information

### Comment

Phenoxyethyl benzoate has applications in the synthesis of e.g. oxazoles, imidazoles and benzoxazepines (Huang *et al.*, 1996; Gandhi *et al.*, 1995). Esters are also useful as photo-removable protecting groups for carboxylic acids in organic synthesis and biochemistry (Ruzicka *et al.*, 2002; Litera *et al.*, 2006; Sheehan & Umezaw, 1973). Keeping this in view, the title compound, C_15_H_14_O_3_, was synthesized and its crystal structure is repored herein.

In the title compound (Fig. 1), the dihedral angle between the benzene rings is 75.85 (7)°. In the crystal, two centrosymmetrically related molecules are weakly associated through a pair of intermolecular interactions between a benzene ring [C1–C6] and an oxygen of the phenoxy group (O1*A*) [ring centroid(*Cg*)···O1*A* separation, 3.912 (7) Å] [for symmetry code (*A*): -*x*, -*y* + 1, -*z*]. In addition, the molecules are weakly associated through a similar pair of intermolecular interactions between the second benzene ring [C10–C15] and a carboxyl oxygen of the ester group (O2*B*) [ring centroid (*Cg*)···O2*B* separation, 3.952 (7) Å] [for symmetry code (*B*): -*x* + 1, -*y*, -*z*] (Fig. 2). The result is a chain structure extending across [110].

### Experimental

Benzoic acid (10.0 g, 0.08 mol) was mixed directly without solvent with 2-phenoxyethanol (11.0 g, 0.08 mol) and refluxed for 3 hours. The reaction was left at room temperature for 24 hours. The product was collected as crystals in 52% yield. The crystals were purified by washing several times with cold ethanol.

### Refinement

Hydrogen atoms were positioned geometrically with C—H = 0.93 Å (aromatic) or 0.98 Å (methylene) and allowed to ride in the refinement, with *U*_iso_(H) = 1.2*U*_eq_(C).

### Crystal data

**Table:**

C_15_H_14_O_3_	*F*(000) = 512
*M**_r_* = 242.26	*D*_x_ = 1.253 Mg m^−^^3^
Monoclinic, *P*2_1_/*c*	Mo *K*α radiation, λ = 0.71073 Å
Hall symbol: -P 2ybc	Cell parameters from 1664 reflections
*a* = 9.4675 (10) Å	θ = 3.0–29.3°
*b* = 10.1411 (10) Å	µ = 0.09 mm^−^^1^
*c* = 13.7792 (12) Å	*T* = 293 K
β = 103.895 (10)°	Wedge, colourless
*V* = 1284.2 (2) Å^3^	0.32 × 0.26 × 0.18 mm
*Z* = 4	

### Data collection

**Table:**

Oxford Diffraction Xcalibur Eos CCD-detector diffractometer	2269 independent reflections
Radiation source: Enhance (Mo) X-ray Source	1479 reflections with *I* > 2σ(*I*)
Graphite monochromator	*R*_int_ = 0.029
Detector resolution: 16.0534 pixels mm^-1^	θ_max_ = 25.0°, θ_min_ = 3.0°
ω scans	*h* = −11→10
Absorption correction: analytical (*CrysAlis PRO*; Agilent, 2011)	*k* = −9→12
*T*_min_ = 0.995, *T*_max_ = 0.997	*l* = −12→16
5089 measured reflections	

### Refinement

**Table:**

Refinement on *F*^2^	Secondary atom site location: difference Fourier map
Least-squares matrix: full	Hydrogen site location: inferred from neighbouring sites
*R*[*F*^2^ > 2σ(*F*^2^)] = 0.044	H-atom parameters constrained
*wR*(*F*^2^) = 0.130	*w* = 1/[σ^2^(*F*_o_^2^) + (0.0531*P*)^2^ + 0.042*P*] where *P* = (*F*_o_^2^ + 2*F*_c_^2^)/3
*S* = 1.02	(Δ/σ)_max_ < 0.001
2269 reflections	Δρ_max_ = 0.11 e Å^−^^3^
164 parameters	Δρ_min_ = −0.11 e Å^−^^3^
0 restraints	Extinction correction: *SHELXL97* (Sheldrick, 2008), Fc^*^=kFc[1+0.001xFc^2^λ^3^/sin(2θ)]^-1/4^
Primary atom site location: structure-invariant direct methods	Extinction coefficient: 0.035 (3)

### Special details

**Table:**

Geometry. All esds (except the esd in the dihedral angle between two l.s. planes) are estimated using the full covariance matrix. The cell esds are taken into account individually in the estimation of esds in distances, angles and torsion angles; correlations between esds in cell parameters are only used when they are defined by crystal symmetry. An approximate (isotropic) treatment of cell esds is used for estimating esds involving l.s. planes.
Refinement. Refinement of F^2^ against ALL reflections. The weighted R-factor wR and goodness of fit S are based on F^2^, conventional R-factors R are based on F, with F set to zero for negative F^2^. The threshold expression of F^2^ > 2sigma(F^2^) is used only for calculating R-factors(gt) etc. and is not relevant to the choice of reflections for refinement. R-factors based on F^2^ are statistically about twice as large as those based on F, and R- factors based on ALL data will be even larger.

### Fractional atomic coordinates and isotropic or equivalent isotropic displacement parameters (Å^2^)

**Table:**

	*x*	*y*	*z*	*U*_iso_*/*U*_eq_	
O2	0.21934 (14)	0.06786 (14)	0.04977 (8)	0.0697 (5)	
O1	0.10626 (13)	0.31548 (14)	−0.04881 (9)	0.0693 (4)	
C1	0.0173 (2)	0.39845 (19)	−0.11534 (12)	0.0573 (5)	
C10	0.37587 (19)	−0.11334 (19)	0.06159 (13)	0.0589 (5)	
C9	0.3215 (2)	−0.0031 (2)	0.11219 (14)	0.0644 (5)	
C7	0.0433 (2)	0.2408 (2)	0.01671 (14)	0.0703 (6)	
H7A	−0.0199	0.1734	−0.0206	0.084*	
H7B	−0.0148	0.2977	0.0484	0.084*	
C8	0.1615 (2)	0.1781 (2)	0.09424 (14)	0.0786 (6)	
H8A	0.2379	0.2417	0.1196	0.094*	
H8B	0.1232	0.1479	0.1497	0.094*	
O3	0.36163 (17)	0.02131 (18)	0.19998 (10)	0.0968 (6)	
C2	−0.1322 (2)	0.4027 (2)	−0.13240 (14)	0.0683 (6)	
H2A	−0.1804	0.3477	−0.0970	0.082*	
C5	0.0077 (3)	0.5679 (2)	−0.23750 (14)	0.0793 (7)	
H5A	0.0554	0.6235	−0.2728	0.095*	
C6	0.0870 (2)	0.4820 (2)	−0.16825 (14)	0.0709 (6)	
H6A	0.1879	0.4800	−0.1569	0.085*	
C11	0.4723 (2)	−0.2012 (2)	0.11906 (17)	0.0810 (7)	
H11A	0.5006	−0.1905	0.1881	0.097*	
C15	0.3333 (2)	−0.1315 (2)	−0.04026 (15)	0.0737 (6)	
H15A	0.2669	−0.0739	−0.0794	0.088*	
C3	−0.2097 (2)	0.4898 (2)	−0.20273 (16)	0.0809 (7)	
H3A	−0.3107	0.4923	−0.2147	0.097*	
C4	−0.1402 (3)	0.5723 (2)	−0.25494 (15)	0.0808 (7)	
H4A	−0.1934	0.6307	−0.3018	0.097*	
C13	0.4865 (3)	−0.3199 (3)	−0.0267 (2)	0.1029 (8)	
H13A	0.5257	−0.3886	−0.0564	0.123*	
C14	0.3890 (3)	−0.2350 (2)	−0.08458 (18)	0.0943 (7)	
H14A	0.3603	−0.2471	−0.1534	0.113*	
C12	0.5261 (3)	−0.3035 (3)	0.0747 (2)	0.1039 (9)	
H12A	0.5904	−0.3628	0.1138	0.125*	

### Atomic displacement parameters (Å^2^)

**Table:**

	*U*^11^	*U*^22^	*U*^33^	*U*^12^	*U*^13^	*U*^23^
O2	0.0937 (10)	0.0559 (9)	0.0580 (7)	0.0144 (8)	0.0151 (7)	0.0017 (7)
O1	0.0707 (8)	0.0666 (9)	0.0747 (8)	0.0111 (7)	0.0255 (7)	0.0141 (7)
C1	0.0673 (12)	0.0505 (12)	0.0551 (10)	0.0079 (10)	0.0163 (9)	−0.0068 (9)
C10	0.0561 (11)	0.0540 (12)	0.0664 (12)	−0.0040 (10)	0.0146 (9)	0.0108 (10)
C9	0.0665 (12)	0.0659 (14)	0.0601 (11)	−0.0028 (11)	0.0139 (9)	0.0091 (11)
C7	0.0862 (14)	0.0593 (14)	0.0730 (12)	0.0087 (12)	0.0342 (10)	0.0017 (11)
C8	0.1139 (17)	0.0638 (14)	0.0627 (11)	0.0174 (13)	0.0303 (12)	0.0018 (11)
O3	0.1082 (12)	0.1176 (15)	0.0581 (8)	0.0162 (10)	0.0073 (8)	−0.0040 (9)
C2	0.0685 (13)	0.0602 (14)	0.0758 (12)	0.0002 (11)	0.0164 (10)	−0.0068 (11)
C5	0.1058 (18)	0.0711 (16)	0.0663 (12)	0.0138 (14)	0.0312 (12)	0.0101 (12)
C6	0.0747 (13)	0.0719 (15)	0.0716 (12)	0.0103 (12)	0.0284 (10)	0.0065 (12)
C11	0.0631 (13)	0.0809 (17)	0.0913 (15)	0.0054 (12)	0.0036 (11)	0.0145 (14)
C15	0.0933 (15)	0.0593 (14)	0.0717 (13)	0.0159 (12)	0.0262 (11)	0.0103 (11)
C3	0.0709 (13)	0.0768 (17)	0.0855 (14)	0.0073 (13)	0.0003 (12)	−0.0152 (14)
C4	0.1031 (18)	0.0686 (16)	0.0614 (12)	0.0196 (14)	0.0018 (12)	−0.0062 (11)
C13	0.107 (2)	0.0694 (17)	0.148 (2)	0.0217 (16)	0.0600 (19)	0.0079 (19)
C14	0.126 (2)	0.0727 (17)	0.0922 (15)	0.0136 (16)	0.0415 (14)	0.0034 (14)
C12	0.0790 (16)	0.089 (2)	0.139 (2)	0.0257 (15)	0.0167 (16)	0.0231 (19)

### Geometric parameters (Å, º)

**Table:**

O2—C9	1.339 (2)	C5—C4	1.364 (3)
O2—C8	1.445 (2)	C5—C6	1.374 (3)
O1—C1	1.373 (2)	C5—H5A	0.9300
O1—C7	1.415 (2)	C6—H6A	0.9300
C1—C2	1.379 (2)	C11—C12	1.364 (3)
C1—C6	1.384 (3)	C11—H11A	0.9300
C10—C15	1.376 (2)	C15—C14	1.381 (3)
C10—C11	1.381 (3)	C15—H15A	0.9300
C10—C9	1.474 (3)	C3—C4	1.370 (3)
C9—O3	1.203 (2)	C3—H3A	0.9300
C7—C8	1.491 (3)	C4—H4A	0.9300
C7—H7A	0.9700	C13—C12	1.367 (3)
C7—H7B	0.9700	C13—C14	1.369 (3)
C8—H8A	0.9700	C13—H13A	0.9300
C8—H8B	0.9700	C14—H14A	0.9300
C2—C3	1.384 (3)	C12—H12A	0.9300
C2—H2A	0.9300		
			
C9—O2—C8	115.63 (14)	C4—C5—H5A	119.7
C1—O1—C7	117.97 (14)	C6—C5—H5A	119.7
O1—C1—C2	124.94 (18)	C5—C6—C1	120.2 (2)
O1—C1—C6	115.68 (17)	C5—C6—H6A	119.9
C2—C1—C6	119.39 (18)	C1—C6—H6A	119.9
C15—C10—C11	119.3 (2)	C12—C11—C10	120.0 (2)
C15—C10—C9	122.27 (17)	C12—C11—H11A	120.0
C11—C10—C9	118.41 (18)	C10—C11—H11A	120.0
O3—C9—O2	122.7 (2)	C10—C15—C14	120.2 (2)
O3—C9—C10	124.74 (18)	C10—C15—H15A	119.9
O2—C9—C10	112.59 (16)	C14—C15—H15A	119.9
O1—C7—C8	109.06 (16)	C4—C3—C2	121.1 (2)
O1—C7—H7A	109.9	C4—C3—H3A	119.5
C8—C7—H7A	109.9	C2—C3—H3A	119.5
O1—C7—H7B	109.9	C5—C4—C3	119.3 (2)
C8—C7—H7B	109.9	C5—C4—H4A	120.3
H7A—C7—H7B	108.3	C3—C4—H4A	120.3
O2—C8—C7	108.78 (15)	C12—C13—C14	120.0 (3)
O2—C8—H8A	109.9	C12—C13—H13A	120.0
C7—C8—H8A	109.9	C14—C13—H13A	120.0
O2—C8—H8B	109.9	C13—C14—C15	119.7 (2)
C7—C8—H8B	109.9	C13—C14—H14A	120.1
H8A—C8—H8B	108.3	C15—C14—H14A	120.1
C1—C2—C3	119.3 (2)	C11—C12—C13	120.7 (2)
C1—C2—H2A	120.3	C11—C12—H12A	119.7
C3—C2—H2A	120.3	C13—C12—H12A	119.7
C4—C5—C6	120.7 (2)		
			
C7—O1—C1—C2	−8.7 (3)	O1—C1—C6—C5	179.26 (17)
C7—O1—C1—C6	171.67 (16)	C2—C1—C6—C5	−0.3 (3)
C8—O2—C9—O3	1.1 (3)	C15—C10—C11—C12	1.0 (3)
C8—O2—C9—C10	−179.74 (16)	C9—C10—C11—C12	−179.3 (2)
C15—C10—C9—O3	−175.5 (2)	C11—C10—C15—C14	−1.3 (3)
C11—C10—C9—O3	4.7 (3)	C9—C10—C15—C14	178.9 (2)
C15—C10—C9—O2	5.3 (3)	C1—C2—C3—C4	−0.5 (3)
C11—C10—C9—O2	−174.41 (17)	C6—C5—C4—C3	−0.2 (3)
C1—O1—C7—C8	−169.37 (15)	C2—C3—C4—C5	0.3 (3)
C9—O2—C8—C7	−176.41 (17)	C12—C13—C14—C15	1.3 (4)
O1—C7—C8—O2	−75.6 (2)	C10—C15—C14—C13	0.2 (4)
O1—C1—C2—C3	−179.05 (17)	C10—C11—C12—C13	0.5 (4)
C6—C1—C2—C3	0.5 (3)	C14—C13—C12—C11	−1.7 (4)
C4—C5—C6—C1	0.2 (3)		
